# Activation mechanism of human soluble guanylate cyclase by stimulators and activators

**DOI:** 10.1038/s41467-021-25617-0

**Published:** 2021-09-17

**Authors:** Rui Liu, Yunlu Kang, Lei Chen

**Affiliations:** 1 State Key Laboratory of Membrane Biology, College of Future Technology, Institute of Molecular Medicine, Beijing Key Laboratory of Cardiometabolic Molecular Medicine, Peking University, 100871 Beijing, China; 2 Peking-Tsinghua Center for Life Sciences, Peking University, 100871 Beijing, China; 3 Academy for Advanced Interdisciplinary Studies, Peking University, 100871 Beijing, China

**Keywords:** Enzymes, Cryoelectron microscopy, Cardiovascular biology

## Abstract

Soluble guanylate cyclase (sGC) is the receptor for nitric oxide (NO) in human. It is an important validated drug target for cardiovascular diseases. sGC can be pharmacologically activated by stimulators and activators. However, the detailed structural mechanisms, through which sGC is recognized and positively modulated by these drugs at high spacial resolution, are poorly understood. Here, we present cryo-electron microscopy structures of human sGC in complex with NO and sGC stimulators, YC-1 and riociguat, and also in complex with the activator cinaciguat. These structures uncover the molecular details of how stimulators interact with residues from both β H-NOX and CC domains, to stabilize sGC in the extended active conformation. In contrast, cinaciguat occupies the haem pocket in the β H-NOX domain and sGC shows both inactive and active conformations. These structures suggest a converged mechanism of sGC activation by pharmacological compounds.

## Abstract (web summary)

Soluble guanylate cyclase (sGC) is a validated drug target for cardiovascular diseases. Here, the authors report structures of human sGC in complex with NO and sGC stimulators or activator, providing insight into the mechanism of sGC activation by pharmacological compounds.

## Introduction

Nitric oxide (NO) signaling plays an essential role in many physiological processes, and deregulation of NO signaling can lead to a spectrum of diseases^1–4^. NO signaling is initiated by the activation of NO synthase in the donor cell to produce NO molecules. NO then readily crosses target cell membranes and binds to its primary receptor soluble guanylate cyclase (sGC), which in turn boosts the activity of sGC several hundred-fold to produce intracellular cGMP^2,5^.

sGC is a heterodimer composed of one α and one β subunit. The α and β subunits share sequence homology and a common domain arrangement: the N-terminal H-NOX and PAS domains, the middle CC domain, and the C-terminal catalytic domain^6^. Recent high-resolution structures of human sGC in different functional states have revealed that sGC is an allosteric enzyme that is composed of three structural modules: the sensor module, the transducer module, and the catalytic module^7^. Saturated NO binding to sGC drives the conformational change of the sensor module. This signal is conveyed to the adjacent transducer module and is further allosterically transmitted to the catalytic module to open the GTP binding cleft and enhance its activity^7^. The conformational changes within each module during sGC activation are associated with the large overall structural reconfiguration of the whole enzyme: inactive sGC has a bent shape, while active sGC is extended^7^. Similar structural changes were also observed in the medium-resolution structures of sGC from insect *Manduca sexta*^8^, further emphasizing the evolutionarily conserved structural mechanism of sGC activation by NO. The H-NOX domain of the β subunit in the sensor module harbors a prosthetic ferrous haem (Fe^2+^) moiety, which is essential for NO sensing^9^. In vitro structural and biochemical studies showed the oxidization of ferrous haem into ferric haem (Fe^3+^) can trap sGC in the inactive state^10–12^. Moreover, the ferric haem can easily dissociate from sGC; this results in haem-free sGC, which has low activity and responds poorly to NO stimulation^13^. The haem oxidization and dissociation processes have been observed in vivo as well and can lead to the attenuation of NO signaling in several pathological conditions, such as in inflammation or under oxidative stress^14,15^.

To enhance the downstream of NO signaling by increasing the sGC activity in related diseases, two types of small pharmaceutical molecules have been developed, namely, sGC stimulators and activators. sGC stimulators can activate sGC with ferrous haem without NO, and also have strong synergistic activation effect with NO. Therefore, sGC stimulators can enhance downstream cGMP signaling both in the absence and in the presence of NO^4,16,17^. The sGC stimulator riociguat is clinically used for the treatment of pulmonary arterial hypertension and chronic thromboembolic pulmonary hypertension^18^, and another stimulator, vericiguat, was developed for the treatment of symptomatic chronic heart failure with reduced ejection fraction^19^. On the other hand, sGC activators can bind to and activate the haem-oxidized and haem-free sGC irrespective of the upstream NO signaling. They are used to activate sGC where impaired NO signaling is caused by haem oxidization and the subsequent loss of haem in sGC^16,17^. Despite their functional importance, the structural mechanisms through which sGC stimulators and activators bind and activate sGC at high resolution have been unknown for decades, especially in the context of full-length human sGC. Here we investigated the structures of human α1β1 sGC in complex with the stimulator YC-1 and riociguat and the activator cinaciguat by cryo-electron microscopy (cryo-EM). These structures not only reveal the detailed interactions involved in sGC–ligand recognition but also uncover the structural changes associated with drug binding and enzyme activation.

## Results

### Structure of sGC in complex with YC-1 or riociguat in the presence of NO

**Fig. 1:**
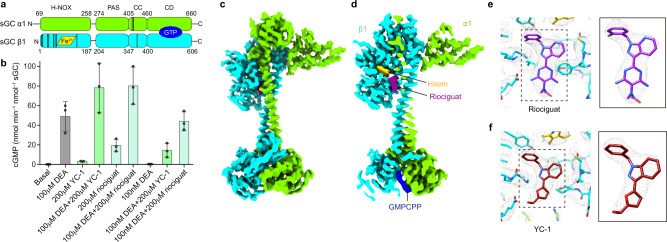
Cryo-EM structure of sGC in the presence of NO and stimulators. **a** Domain organization of the human α1β1 sGC heterodimer. The haem cofactor and the GTP substrate-binding site are shown in yellow and blue, respectively. The positions of residues interacting with riociguat are indicated with vertical dark lines. **b** End-point activity assay of wild-type sGC in the presence or absence of DEA, YC-1, or riociguat as indicated. Mean ± s.d., *n* = 3 biologically independent reactions. Source data are provided as a Source Data file. **c** Cryo-EM map of sGC in complex with NO and riociguat. The densities of haem, riociguat, and GMPCPP are shown in yellow, purple, and blue, respectively. **d** The cut-open view of (**c**). **e** Density at the riociguat–binding site. The map is shown as a gray mesh and atomic model is shown as sticks. **f** Density at the YC-1–binding site. The map is shown as a gray mesh and atomic model is shown as sticks.

YC-1 is a prototype sGC stimulator^20^, and based on its chemical structure, a series of stimulators, such as BAY41-2272, BAY41-8543, riociguat (BAY 63-2521), and vericiguat (BAY 1021189), have been developed (Supplementary Fig. 1a). Among them, riociguat exhibits high potency, and good drug metabolism and pharmacokinetic properties and has been approved for clinical use^21^. Previous studies suggested YC-1 might bind to the α1 H-NOX domain^22,23^, the catalytic module^24–27^, or the β1 H-NOX domain^28–30^. A recent cryo-EM map of *M.sexta* sGC at 5.8 Å resolution in the presence of YC-1 and NO revealed an extra density located between the β H-NOX and CC domains^8^. This extra density was suggested to be the putative YC-1 molecule. However, the exact binding pose of YC-1 and residues on sGC that interact with YC-1 could not be determined due to the limited resolution. Moreover, it is still questionable whether this site is the functional stimulatory site for human sGC, since no associated mutagenesis data were available. To directly identify the stimulator-binding site at high resolution, we embarked structural studies using cryo-EM. As the first step, we confirmed that purified human α1β1 sGC can be activated by the stimulator YC-1 and riociguat (Fig. 1 and Supplementary Fig. 1). The activation of sGC by stimulators and NO shows strong synergistic effects (Fig. 1b)^31,32^, suggesting that both the stimulators and NO promote sGC to adopt the same biochemically active state. Therefore, we prepared the cryo-EM sample of full-length human α1β1 sGC in complex with YC-1 or riociguat in the presence of saturated NO donor. The final reconstruction reached a resolution of 3.9 Å and 3.7 Å in the presence of YC-1 and riociguat, respectively (Fig. 1c–f, Supplementary Figs. 2 and 3, and Supplementary Table 1).

The structures of sGC in complex with NO and stimulators show an extended conformation (Fig. 1c and d), similar to the NO-activated state, with a root-mean-square deviation (RMSD) of 0.8 Å between the NO + YC-1 structure and the NO-activated state structure (PDB ID: 6JT2)^7^. We observed the haem-H105 bonds were broken in these structures (Supplementary Fig. 2m). The cryo-EM density maps revealed that YC-1 and riociguat bind at the same site on sGC, similar to the site observed in *M.sexta* sGC^8^. The stimulator-binding site is located between the β H-NOX and CC domains (Fig. 1e and f), in agreement with their similar chemical structures. The local map qualities are sufficient for us to model YC-1 and riociguat molecules (Fig. 1e and f).

### The stimulator-binding site

**Fig. 2:**
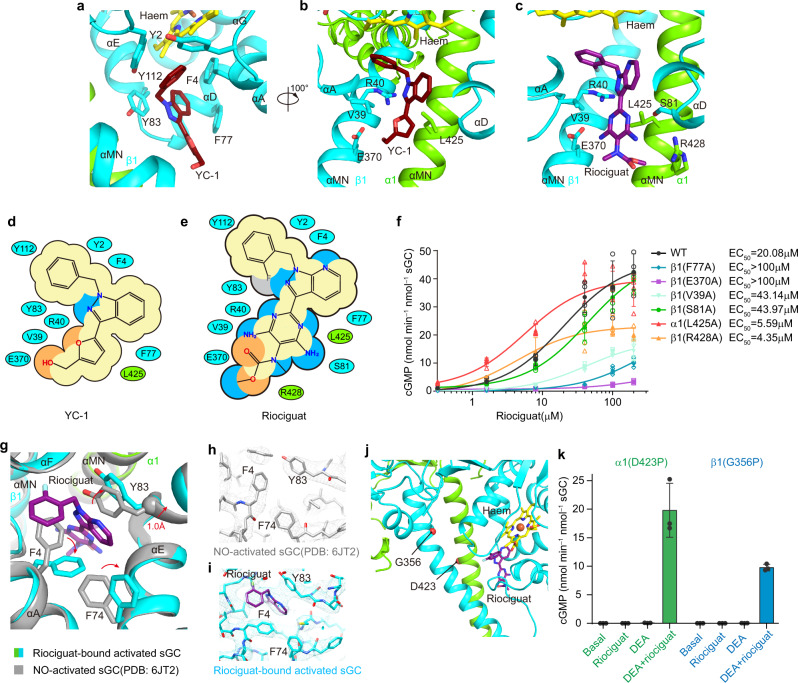
Binding site of sGC stimulators. **a** The interactions between YC-1 and sGC. Protein is colored the same as in Fig. 1. **b** A 100° rotated view compared to (**a**). **c** The interactions between riociguat and sGC in the same view as in (**b**). **d** Cartoon representation of interactions between YC-1 and sGC. Residues from the α1 and β1 subunits are shown as green and cyan, respectively. **e** Cartoon representation of interactions between riociguat and sGC. Residues are colored the same as in (**d**). **f** Dose-dependent activation curve of wild type and alanine mutants of sGC in the presence of 1 mM BIC and riociguat at different concentrations ranged from 0.32 mM to 200 mM. Mean ± s.d., *n* = 3 or 4 independent reactions. Source data are provided as a Source Data file. **g** Structure comparison between NO- and riociguat- bound states (colored) and the NO-activated state (gray) at the stimulator-binding site. The conformational changes induced by riociguat binding are indicated by red arrows. **h**–**i** Cryo-EM density maps around the sGC stimulators binding site in the NO-activated state (**h**) and NO- and riociguat- bound state (**i**). The residues undergo conformational changes in the two states shown in (**g**) are labeled. **j** Locations of the α1 D423 and β1 G356 residues in the NO- and riociguat- bound state. The aC atoms of α1 D423 and β1 G356 are shown as red spheres. **k** End-point activity assay of sGC proline mutations in the absence or presence of 100 mM DEA or 200 mM riociguat. Mean ± s.d., *n* = 3 independent reactions. Source data are provided as a Source Data file.

YC-1 is clamped in the cleft between the N- and C-terminal subdomains of the β1 H-NOX and CC domains (Fig. 2). The side chain of β1 Y112 stacks with the terminal phenyl ring of YC-1, which also makes hydrophobic interactions with the side chain of β1 Y2, F4, and haem (Fig. 2a and d). The central indazole moiety of YC-1 interacts with Y83, F77, and F4 of the β1 H-NOX domain (Fig. 2a, b, and d). The furan group of YC-1 interacts with β1 V39, β1 R40, and α1 L425 (Fig. 2b and d). The terminal hydroxyl group of YC-1 makes hydrogen bonds with β1 E370 on the CC domain (Fig. 2b and d). Compared with YC-1, the core of riociguat binds to sGC in a similar way (Fig. 2c and e), but the newly introduced diaminopyrimidine group makes additional polar interactions with β1 S81 (Fig. 2c and e) and the terminal methylcarbamate moiety is close to α1 R428 (Fig. 2c and e). We next examined the effects of riociguat on various stimulator-binding site mutants in the presence of saturated butyl isocyanide (BIC), which is a distal haem ligand and partial agonist for sGC^33^ (Fig. 2f and Supplementary Fig. 6i). We chose BIC instead of NO donors, because sGC can be dose-dependently activated by riociguat in the presence of saturated BIC (1 mM) with an EC_50_ value of 20.08 µM (Fig. 2f and Supplementary Fig. 6i) and BIC concentration in the reaction system is easy to control, while NO release from NO donors, such as DEA NONOate, heavily depends on pH, temperature and incubation time and therefore NO concentration is difficult to control accurately, especially in an aerobic environment. We found mutations of F77, E370 and V39 on β1 subunit, all of which interact with the common structural features of YC-1–type stimulators, into alanine residues reduced the potency of riociguat to different extent (Fig. 2f and Supplementary Table 2). Mutation of S81 on the β1 subunit that interacts with the tail of riociguat moderately reduced the potency of riociguat. Because S81 only interact with riociguat but not YC-1 (Fig. 2d, e), we speculate the interaction between S81 and the tail of riociguat might play a role in the enhanced potency of riociguat compared with YC-1. BAY41-2272 and BAY41-8543 are additional YC-1–type sGC stimulators that can activate sGC with EC_50_ values at the micromolar range^23,34^ (Supplementary Fig. 1a). Our structures suggest these stimulators bind sGC in a similar pose compared to riociguat and their distinct tails might dictate their different potency. In addition, we found mutations of L425A and R428A on α1 subunit slightly increased the potency of riociguat. Because L425 and R428 are on the same side of the diaminopyrimidine group and close to the bulky diaminopyrimidine-methylcarbamate tail of riociguat (Fig. 2e), we speculate truncation of L425 and R428 to alanines would leave more space at this region so that riociguat might adopt a more favorable conformation to engage with other interacting residues on sGC. These data also suggest further structure-based modification of chemical groups on the tail of riociguat might result in improved molecules that bind sGC with higher potency.

By superimposing the sGC structure in complex with stimulators and NO onto the structure of the NO-activated state (PDB ID: 6JT2) (Fig. 2g)^7^, we found that there are several sterical clashes between the stimulator molecules and the β1 H-NOX domain of the NO-activated state (Fig. 2g). The binding of riociguat pushes αE away from the stimulator-binding site. The Cα atom of Y83 shows a 1.0 Å shift (Fig. 2g). Moreover, the rotamer conformations of several residues are altered as well. The phenyl ring of F4 swings away from the stimulator-binding site and the adjacent F74 shows a concomitant movement (Fig. 2g–i). These conformational changes collectively reshape the stimulator-binding pocket, which does not exist in the NO-activated structure, suggesting the stimulators bind sGC through an induced-fit mechanism. Furthermore, the stimulator binds beside the haem cofactor and might perturb the haem environment (Fig. 2a–c), which explains why YC-1 shifts the Soret peak positions of the sGC-CO complex^35^, changes the Fe-CO stretching frequency in the Raman spectrum^28^, and increases the affinity of CO for the *M.sexta* NT construct^36^. Because the stimulators bind to a site co-formed by the β1 H-NOX and CC domains (Figs. 1 and 2) which are spatially separated in the bent inactive conformation^7^, we propose the binding of stimulators facilitates the activating interactions between β1 H-NOX and CC domains and thus stabilizes sGC in the extended conformation for activation. This agrees with the positive cooperativity between the stimulators and NO. To further support this, we exploited the previously identified proline mutations in the hinge region of CC domains (α1 D423P or β1 G356P) that destabilize the long helices of the transducer module in the extended active conformation due to reduced main chain hydrogen bonding^7^. We reasoned that these mutations might inhibit the transmission of the NO-activation signal from sensor module to transducer module and therefore might elevate the free energy threshold for sGC activation and shift the inactive-active state equilibrium toward the inactive state. Indeed, although NO can bind onto these mutants^7^, the activation effect of NO was impaired by these mutations (Fig. 2k and Supplementary Fig. 6j). Because stimulators bind sGC in the extended conformation, these mutants might allosterically reduce the binding of stimulators. Conversely, once bound, stimulators might stabilize the CC domain of these mutants in the extended conformation to some extent, since the positions of these proline mutations are either above or at the same level of the stimulator-binding site on sGC (Fig. 2j). We found riociguat alone failed to activate either α1 D423P or β1 G356P mutant (Fig. 2k and Supplementary Fig. 6j), likely due to reduced binding of riociguat to these mutants. However, the simultaneous application of NO and riociguat markedly boosted their activity (Fig. 2k and Supplementary Fig. 6j), likely because the free energy contributed by simultaneous binding of NO and riociguat onto sGC overcomes the elevated free energy threshold for enzyme activation of these two mutants, further highlighting the strong synergistic effect of NO and riociguat.

### Structure of sGC in complex with the activator cinaciguat

**Fig. 3:**
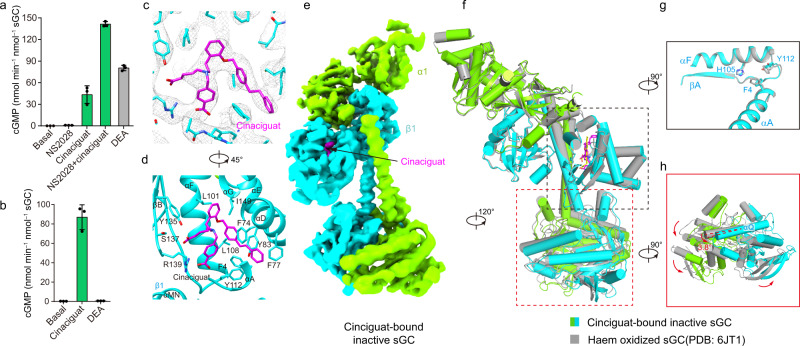
Structure of sGC in the cinaciguat-bound inactive state. **a** End-point activity assay of the wild-type sGC samples in the presence or absence of 20 mM NS2028, 20 mM cinaciguat or 100 mM DEA. NS2028 oxidizes the ferrous haem in sGC to ferric haem. Mean ± s.d., *n* = 3 biologically independent reactions. Source data are provided as a Source Data file. **b** End-point activity assay of the haem-free sGC samples with or without 20 mM cinaciguat or 100 mM DEA as indicated. Mean ± s.d., *n* = 3 biologically independent reactions. Source data are provided as a Source Data file. **c** Density at the cinaciguat binding pocket in the CI state. Cinaciguat is shown as magenta sticks. **d** The interactions between cinaciguat and sGC in the CI state. A 45° rotated view compared to (**c**). **e** Cryo-EM map of sGC in the cinaciguat bound inactive (CI) state. The density of cinaciguat is shown in magenta. **f** Structural comparison of sGC in the CI state (colored) and haem-oxidized state (gray) by aligning the β1 H-NOX domain. **g** A 90° rotated zoom-in view of the β1 H-NOX domain boxed in f (black rectangle). The side chains of H105, Y112, and F4 are shown as sticks. **h** A 90° rotated view of catalytic module boxed in f (red rectangle). The angle between the αQ helices is shown.

sGC activators, represented by cinaciguat (BAY58-2667) (Supplementary Fig. 6a), bind and activate haem-oxidized or haem-free sGC^37^. Although previous studies on the structure of cinaciguat in complex with H-NOX domain of symbiotic cyanobacteria *Nostoc sp* provided valuable insights into the mechanism of cinaciguat binding^38^, the *Nostoc* H-NOX has only ~35% sequence identity to human sGC and its NO-binding properties are largely different from sGC either^39^. To elucidate the binding and activation mechanism of human sGC in the context of full-length enzyme, we prepared sGC protein in complex with cinaciguat through two approaches for cryo-EM studies: in the first approach, we oxidized ferrous sGC protein using NS2028 (8-Bromo-1H,4H-[1,2,4]oxadiazolo[3,4-c][1,4]benzoxazin-1-one), a reagent that can oxidize ferrous haem to ferric haem^11^, and used cinaciguat to replace the oxidized haem (Fig. 3a)^40^; in the second approach, we added cinaciguat to the purified haem-free sGC protein obtained by detergent treatment (Fig. 3b and Supplementary Fig. 4)^41^. Unexpectedly, we found that not only the bent but also the extended conformations existed in both cryo-EM samples, and therefore we merged these data for image processing (Supplementary Fig. 5). The cryo-EM reconstruction of bent sGC and extended sGC reached resolutions of 4.1 Å and 3.8 Å after consensus refinement and 3.9 Å and 3.6 Å at the N-lobe after multibody refinement (Supplementary Fig. 5). The map qualities were sufficient to locate the cinaciguat molecules inside the haem pocket of the β1 H-NOX domain in both maps (Supplementary Fig. 5).

### The cinaciguat-bound inactive structure

The overall shape of the cinaciguat-bound bent structure is similar to that of the inactive state (PDB ID: 6JT1)^7^ (Fig. 3). Cinaciguat binds inside the haem pocket of the β1 H-NOX domain (Fig. 3c and d). The benzoic acid group of cinaciguat interacts with positively charged R139 and the carboxybutyl group of cinaciguat makes hydrogen bonds with Y135 and S137 (Fig. 3d). The two middle phenyl rings of cinaciguat interact with hydrophobic residues in the haem pocket, including I149, L101, L108, and F74 (Fig. 3d). The terminal benzyl group of cinaciguat points to the outside of sGC and is surrounded by the aromatic F4 on αA, F77 on αD, Y83 on the αD-αE loop, and Y112 on αF (Fig. 3d). In comparison with the inactive sGC structure (PDB ID: 6JT1)^7^ (Fig. 3f), the replacement of haem by cinaciguat causes rotamer changes of F4 and Y112 which further leads to small but noticeable structural shifts of αF and βA (residues 100–117) and a slightly altered conformation of the sensor module (Fig. 3g). These changes were amplified by the transducer module into a displacement of the catalytic module as a rotation of about 3.8° (Fig. 3h). However, the GTP-binding site in the catalytic module is still incompatible with substrate binding and is devoid of substrate analog GMPCPP density (Supplementary Fig. 6b), confirming that this structure represents an inactive state. We name this structure as the cinaciguat-bound inactive (CI) state, in which the activator is bound but does not exert its activating function.

### The cinaciguat-bound activated structure

**Fig. 4:**
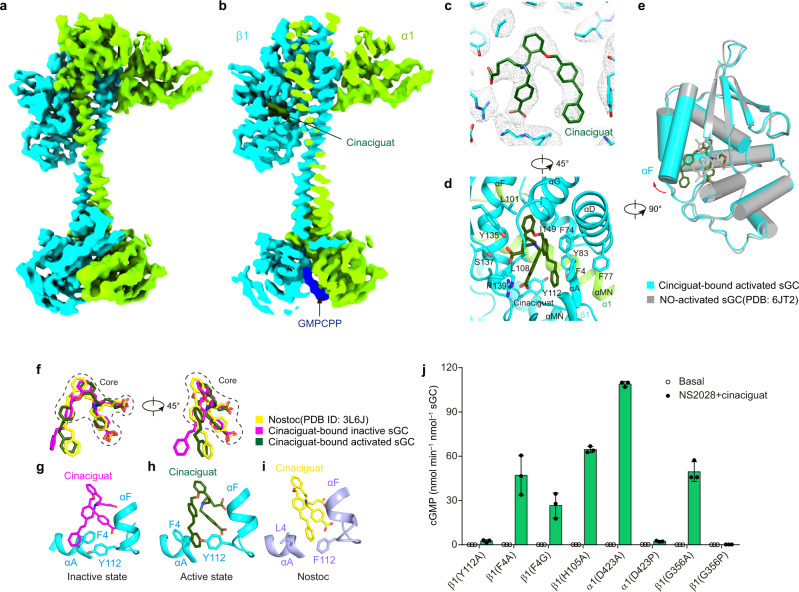
Structure of sGC in the cinaciguat-bound activated state. **a** Cryo-EM map of sGC in the cinaciguat-bound activated state (CA) state. The density of cinaciguat is shown in green. **b** The cut-open view of (**a**). **c** Density at the cinaciguat-binding site in CA state. Cinaciguat is shown as dark green sticks. **d** The interactions between cinaciguat and sGC. A 45° rotated view compared to (**b**). **e** Structure comparison of sGC β1 H-NOX in the CA state (colored) and the NO-activated state (gray). **f** Structural comparison of cinaciguat in the Nostoc H-NOX domain (PDB ID: 3L6J, yellow), CI state (magenta) and CA state (green). The core of cinaciguat is indicated by dashes. **g** The interactions between the terminal benzyl group of cinaciguat and F4 and Y112 of sGC in the CI state. **h** The interactions between the terminal benzyl group of cinaciguat and F4 and Y112 of sGC in the CA state. **i** The interaction between the terminal benzyl group of cinaciguat and L4 and F112 of the Nostoc H-NOX domain (PDB ID: 3L6J). **j** End-point activity assay of the various sGC mutants in the presence or absence of 20 mM NS2028 and 20 mM cinaciguat as indicated. Mean ± s.d., *n* = 3 biologically independent reactions. Source data are provided as a Source Data file.

In the cinaciguat-bound extended conformation (Fig. 4a and b), cinaciguat interacts with a similar set of residues compared to the CI state (Fig. 4c and d). However, it induces a much larger conformational change of the β1 H-NOX domain and leads to the complete extension of sGC (Fig. 4). The RMSD between this structure and the NO-activated structure (PDB ID: 6JT2) is 0.39 Å. Moreover, we found the GMPCPP density inside the catalytic module (Fig. 4b), further confirming this is a catalytically active state. Therefore, we name this structure as the cinaciguat-bound activated (CA) state. To understand why cinaciguat-bound sGC can adopt both bent and extended structures, we compared the conformations of cinaciguat in the structures of the H-NOX domain in CI and CA states and the crystal structure of *Nostoc* H-NOX in complex with cinaciguat (PDB ID: 3L6J) (Fig. 4f–i)^38^. We found the interactions between the cinaciguat core and the β1 H-NOX domain are similar in these structures but the conformations of the terminal benzyl group of cinaciguat are dramatically distinct (Fig. 4f–i). This is consistent with the high flexibility of cinaciguat, which has 17 rotatable bonds, and multiple conformations of cinaciguat were previously observed in the NMR structures of the cinaciguat-bound isolated H-NOX domain (PDB ID: 5MNW) (Supplementary Fig. 6c). Although the conformation of β1 H-NOX domain and cinaciguat molecule in both CI and CA states were different from *Nostoc* H-NOX structure (Supplementary Fig. 6e, f), the cinaciguat binds to conserved hydrophilic residues that are involved in haem binding, such as Y135, S137 and R139. However, there are several non-conserved residues between human sGC and *Nostoc* H-NOX domain which determine the conformational difference between them. In the *Nostoc* H-NOX structure, the hydrophobic benzyl group is bound by Y2, F112, and Y83 (Supplementary Fig. 6d), while in the CI and CA structures, the benzyl group makes additional interactions with F4 on αA of human sGC (Fig. 4g and h), whereas a smaller leucine residue at this position in *Nostoc* H-NOX replaces the F4 of the human counterpart (Fig. 4i). Between the CI and CA structures, F4 and Y112 have the largest conformational differences (Fig. 4g and h). To further explore the functions of F4 and Y112 on cinaciguat activation, we mutated these bulky residues into smaller residues. We found mutations of F4 into alanine or glycine moderately reduced the efficacy of cinaciguat-bound sGC, while mutation of Y112A completely abolished the activation by cinaciguat (Fig. 4j). Because Y112 is located on the C terminus of αF, which is important for sGC activation, we propose that cinaciguat in the CA state pushes the C terminus of αF to induce a large conformational change of the sensor module and this signal is transmitted to catalytic module via the central transducer module, which finally leads to the full activation of the whole enzyme. In agreement with this model, cinaciguat failed to activate the α1 D423P or β1 G356P mutants of the CC domain (Fig. 4j and Supplementary Fig. 6g), due to the destabilizing effect of proline mutation at the hinge region of the transducer module^7^. In contrast, cinaciguat in the CI state does not push Y112 far enough for a large conformational change of the sensor module (Fig. 3g), and sGC still adopts an inactive structure. We further showed that cinaciguat could robustly activate the H105A mutant (Fig. 4j), suggesting H105 is not essential for cinaciguat activation. This is different from the activation by NO, which absolutely requires H105 for activation^42,43^. It is reported that extension of the terminal group of cinaciguat resulted in compound 20 with higher efficacy^44^ (Supplementary Fig. 6a), and Y112A reduced the activation effect of compound 20^44^, further highlighting the importance of Y112 for sGC activation by cinaciguat-type activators.

## Discussion

It is well established that NO concentrations are perceived by sGC and converted to cGMP levels for downstream signaling. How sGC senses NO is a fundamental question in the field. It is proposed that stoichiometric NO can partially activate the enzyme, while complete activation of sGC requires excess NO^5^. Recent small angle X-ray scattering results provided low-resolution structural evidence that stoichiometric NO induces a partially active conformation which is in between the bent and the fully-extended conformation^8^, while excess NO renders sGC to predominately adopt the fully-extended conformation^7^. The stimulators, exemplified by the YC-1, can boost the activity of sGC in stoichiometric NO state to the level close to excess NO state^8^. In agreement with this, stimulators (YC-1 and riociguat) markedly enhanced the activity of sGC at low concentration of NO donor with positive co-cooperativity (Fig. 1b). But how this cooperativity is achieved at atomic level was enigmatic. Recent medium-resolution structure of *M.sexta* sGC provided the first clue of where YC-1 binds^8^. Our current studies provide near-atomic resolution depiction of how stimulators are recognized by sGC. The stimulators bind at a unique site that is formed by residues not only from the β1 H-NOX domain of sensor module but also from the transducer module, and stabilize sGC in the extended conformation, intuitively explaining their positive cooperativity with NO. Notably, in order to obtain a homogenous sGC population for structural studies, we saturated sGC protein with excess NO in these cryo-EM samples. But we reasoned the stimulators also bind at the same site in the presence of stoichiometric NO, partial agonist CO or BIC, because on one hand, these ligands showed similar positive cooperativity with stimulators, and on the other hand, mutations in stimulator-binding pocket observed here altered the potency of stimulator in the presence of partial agonist, exemplified by BIC. Unfortunately, due to the small size of NO molecule (only two light atoms) and limited resolutions of the cryo-EM maps we obtained, we cannot directly visualize the densities of NO molecules. However, we observed the shift of Soret peak, markedly enhance sGC activity of our cryo-EM sample and the broken of H105-haem bonds in our cryo-EM maps, suggesting the haem was bound with NO and in 5-coordination state in these structures. The exact binding sites of NO and the structure of sGC in stoichiometric NO state still await further investigation. The conformational changes in the sensor module and the transducer module induced by agonists, stimulators and activators are finally conveyed to the catalytic module via the inter-module connections to open the GTP substrate binding pocket. In addition to the YC-1 type stimulators, there are other sGC stimulators with distinct chemical structures^16^, which might bind sGC at different sites. As some mutations in the catalytic module can enhance the sGC activity^24,45^, it is possible that certain hypothetic molecules can bind inside the catalytic module to open the substrate binding pocket and to activate sGC. Because the conformation of catalytic module is allosterically coupled to the structure of the sensor module^46–48^, these hypothetic molecules might likely induce similar extended conformation of sGC.

In contrast to stimulators, cinaciguat activator binds inside the haem pocket and replaces haem. It is striking that sGC molecules in both CA and CI conformation co-exist in the cinaciguat-bound sGC sample. But it is unknown if the sGC molecules in the CI state were trapped in the inactive conformation or they are in thermodynamic equilibrium with the CA state. Nevertheless, our structures suggest that modifications of the chemical groups of activators might yield next generation activators that can push Y112 or αF more effectively, can increase the population of sGC in the CA state and thus can enhance the efficacy of activators.

**Fig. 5:**
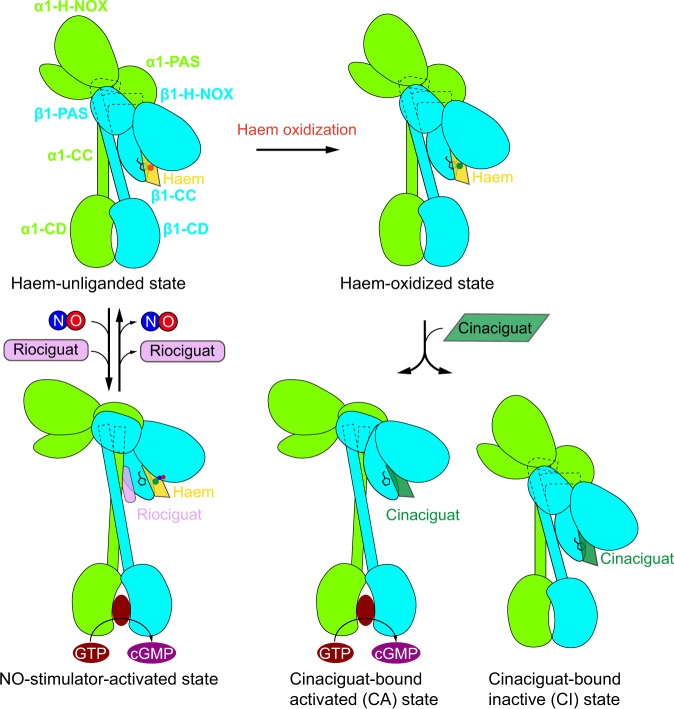
Activation mechanism of sGC by stimulators and activators. Cartoon models of the sGC structures at different functional states. The colors of each individual subunit are the same as Fig. 1a. In the presence of NO, YC-1 type stimulators bind onto sGC with ferrous haem, between β1 H-NOX domain and CC domains, to stabilize sGC in the extended active conformation. Cinaciguat bind onto sGC at the haem-oxidized state or haem-free state, inside the β1 H-NOX domain, and sGC shows both extended active conformation and bent inactive conformation.

In summary, the structures presented here reveal the molecular details of how human α1β1 sGC recognizes pharmaceutical YC-1–type stimulators and cinaciguat–type activators, both of which bind outside of the catalytic module but can allosterically activate the enzyme (Fig. 5). These insights are likely instrumental to future design and optimization of therapeutic drugs that positively modulate the activity of sGC. The structures further strengthen the correlation between the extended conformation of sGC and high enzymatic activity, and also suggest that the bent-to-extended conformational change is a converged activation mechanism for sGC.

## Methods

### Cell culture

Sf9 or Sf21 insect cells (Thermo Fisher Scientific) were cultured in SIM SF (Sino Biological) at 27 °C. The cell lines were routinely checked to be negative for mycoplasma contamination but have not been authenticated.

### Protein expression and purification

To prepare protein sample for cryo-EM studies, supernatants of sf9 or sf21 cells expressing wild-type sGC were loaded onto 2 ml Streptactin Beads 4 FF (Smart-Lifesciences) and washed with W1 buffer (20 mM Tris, pH 8.0, 500 mM NaCl, 2 mM DTT) and W2 buffer(20 mM Tris, pH 8.0, 50 mM NaCl, 2 mM DTT) at 4 °C. Protein was eluted with elution buffer (50 mM Tris, pH 8.5, 50 mM NaCl, 5 mM desthiobiotin, 1 mM EDTA, 2 mM DTT) at 4 °C. The eluate was digested with PreScission protease to remove GFP tag and further purified with a 1 ml HiTrap Q HP column (GE Healthcare) and Superdex 200 Increase (GE Healthcare). For haem-free sGC, fractions eluted from the HiTrap Q HP column (GE Healthcare) were pooled, 10% Tween 20 (final concentration) and PreScission protease were added to the protein, and samples were incubated at 4 °C for about 20 h to remove the haem and GFP tag from the protein. Then the mixture was diluted with buffer A (20 mM Tris, pH 8.0, 2 mM DTT) and then loaded onto a 1 ml HiTrap Q HP column (GE Healthcare) again to separate the protein from impurities. The protein was eluted with buffer B (20 mM Tris, pH 8.0, 500 mM NaCl, 2 mM DTT) at 4 °C in a linear gradient using the AKTA pure system (GE Healthcare). The peak fractions corresponding to the non-tag haem-free sGC were pooled and further purified by Superdex 200 increase (GE Healthcare). The peak fractions containing the sGC were analyzed by 12% SDS-PAGE and were concentrated before flash frozen in liquid nitrogen. The UV-vis spectrums were measured by a spectrometer (Pultton).

To prepare protein sample for enzymatic activity assay, the coding sequence of the sGC α1 subunit tagged with C-terminal GFP-strep and β1 subunit were cloned into the pFastBac1 expression vectors, respectively. The constructs carrying point mutations were generated with Quick Change and corresponding baculoviruses were generated using the Bac-to-Bac system. Two baculoviruses for both α1 and β1 subunits were co-infected into sf9 cells for protein expression. Wild-type sGC and mutants were purified similarly as described above except ion-exchange chromatography was omitted. Protein concentrations were estimated using A280, with molar extinction coefficient of 83.2 mM^−1^ cm^−1^. Haem concentrations were calculated using A431, with molar extinction coefficient of 111 mM^−1^ cm^−1,^^49^. Haem content was the molar ratio between haem and protein.

### Enzymatic activity assay

The purified haem-free sGC were diluted with 20 mM triethanolamine (TEA pH7.6), 300 mM NaCl and subjected to the enzyme activity assay. The reaction system contained 14 nM sGC, 60 mM TEA (pH 7.6), 150 mM NaCl, 0.5 mM DTT, 5 mM MgCl_2_, 200 µM GTP and various chemical drugs as indicated in a final volume of 20 μl. The final concentration of DMSO was 1% (v/v). The assay mixture was incubated at 25 °C for 10 min, and then 80 μl 125 mM Zn(OAc)_2_ and 100 μl 125 mM Na_2_CO_3_ were added to stop the reaction. The GTP-ZnCO_3_ precipitation was removed by centrifuged at 17,000 *g* for 5 min, and the supernatants were used for the quantification of cGMP with the cGMP ELISA Kit (Cayman Chemical) according to the instructions. For activity assay in Figs. 3a, b and 4j, 0.5 mM DTT in the assay mixture was omitted and the reaction temperature was changed to 37 °C. Each assay was repeated for at least three independent reactions.

### Cryo-EM sample preparation and data collection

The purified sGC was concentrated to A_280_ = 4.5 with an estimated concentration of 54 μM. For the sGC in complex with NO and YC-1, 200 µM YC-1 (TargetMol), 1 mM GMPCPP (Biorbyt), 1 mM DEA NONOate (Cayman Chemical), 5 mM MgCl_2_ and 0.5 mM fluorinated octyl-maltoside (FOM, Anatrace) were added to the wild type sGC. For the sGC in complex with NO and riociguat, 200 µM riociguat (TargetMol), 1 mM GMPCPP, 1 mM DEA NONOate, 5 mM MgCl_2_, 0.5 mM FOM were added to the wild type sGC. For the cinaciguat-bound sGC, two approaches were used for the sample preparation: in the first approach, the wild type sGC with ferrous haem were incubated with 200 µM NS2028 and 200 µM cinaciguat (TargetMol) at room temperature overnight, and then 1 mM GMPCPP, 5 mM MgCl_2_, 0.5 mM FOM were added; in the second approach, the purified haem-free sGC was concentrated to A_280_ = 4.5, and then 200 µM cinaciguat, 1 mM GMPCPP, 5 mM MgCl_2_, 0.5 mM FOM were added at 4 °C. Protein samples were loaded onto glow-discharged Quantifoil 0.6/1 holey carbon gold grids and plunged into liquid ethane by Vitrobot Mark IV (Thermo Fisher Scientific). Cryo-grids were screened on a Talos Arctica electron microscope (Thermo Fisher Scientific) operating at 200 kV using a Ceta 16 M camera (Thermo Fisher Scientific). The screened grids were transferred to a Titan Krios electron microscope (Thermo Fisher Scientific) operating at 300 kV with an energy filter set to a slit width of 20 eV. Images were recorded using a K2 Summit direct electron camera (Gatan Inc) in super-resolution mode at a nominal magnification of ×130,000, corresponding to a calibrated super-resolution pixel size of 0.5225 Å. The defocus range was set from −1.5 μm to −1.8 μm. Each image was acquired as a 7.68 s movie stack (32 frames) with a dose rate of 6.25 e^−^Å ^−2^s^−1^, resulting in a total dose of about 48 e^−^Å ^−2^. All data acquisition was done using SerialEM.

### Cryo-EM image analysis

The data processing workflows are shown in Supplementary Figs. 2, 3, 5 and Supplementary Table 1. Super-resolution movie stacks were gain-corrected, motion-corrected, mag-distortion corrected, dose-weighted, and binned to a pixel size of 1.045 Å by MotionCor2 1.1.0 using 5 × 5 patches^50^. Contrast transfer function (CTF) parameters were estimated from non-dose-weighted micrographs using Gctf v1.06^51^. Micrographs with ice or ethane contamination, empty carbon, and poor CTF fit (>5 Å) were manually removed. All classification and reconstruction were performed with Relion 3.0^52^ unless otherwise stated. Particles were picked using Gautomatch (developed by Kai Zhang) and subjected to reference-free 2D classification to remove bad particles. For YC-1 dataset and riociguat dataset, previous human α1β1 sGC map in NO-activated state (EMD-9885) was low-passed to 30 Å and used as initial model for 3D classification. For cinaciguat dataset, four initial models were generated using cryoSPARC^53^ and were used for multi-reference 3D classification in Relion 3.0. The particles selected from good 3D classes were re-centered and re-extracted, and their local CTF parameters were individually determined using Gctf v1.06^51^. These particles were further refined using Relion 3.0 for consensus refinement. We further divided the whole molecule into two bodies—the larger “N lobe” and the smaller “C lobe”—for further multibody refinement in Relion 3.0. The FSC-based resolutions of each iteration during multibody refinement were monitored and we found the first iteration produced best resolution and map features (Supplementary Fig. 2d), probably due to the small size of each body and thus over-fitting during more iterations of multibody refinement. Therefore, we continued the first iteration with “–force_converge” option to generate the final maps of multibody refinement. The two half-maps of each lobe generated by 3D multibody refinement were subjected to post-processing in Relion 3.0. The masked and sharpened maps of each lobe were aligned to the consensus map using UCSF Chimera and summed together to generate the composite map for visualization and interpretation. All of the resolution estimations were based on the Fourier shell correlation (FSC) of 0.143 cutoff after correction of the masking effect^54^. B-factors used for map sharpening were automatically estimated by the post-processing procedure in Relion 3.0. Notably, for cinaciguat-bound dataset, images collected from samples produced by both approaches were found to be similar after initial processing, and therefore were combined for image processing workflow shown in supplementary Fig. 5.

### Model building

The models of human α1β1 sGC^7^ (NO-activated state PDB ID: 6JT2 and heme-oxidized state PDB ID: 6JT1) were placed into the corresponding composite maps using UCSF chimera^55^ and manually rebuilt in Coot^56^. The composite maps were then converted into mtz files and the models were further refined by Phenix^57^ in reciprocal space, and phenix.real_space_refine^57^ and Coot in real space. The resolution of sGC cryo-EM map in riociguat-bound state was not sufficient to explicitly determine the location of the F atom on the phenyl ring, and the F atom was modeled according to the local chemical environment. Figures were made by Chimera X^58^, UCSF chimera and Pymol.

### Quantification and statistical analysis

Global resolution estimations of cryo-EM density maps are based on the 0.143 FSC criterion^54^. The local resolution was estimated using Relion 3.0^52^. The number of independent reactions (N) and the relevant statistical parameters for each experiment (such as mean or standard deviation) are described in the figure legends. No statistical methods were used to pre-determine sample sizes.

### Reporting summary

Further information on research design is available in the Nature Research Reporting Summary linked to this article.

## Supplementary information

supplementary information

Reporting Summary

## Data Availability

The cryo-EM map of sGC in complex with YC1, riociguat and cinaciguat are accessible through the EMDB codes EMD-30619, EMD-30618, EMD-30620 and EMD-30621. The atomic coordinates have been deposited in the Protein Data Bank (PDB) with accession codes 7D9S, 7D9R, 7D9T and 7D9U. PDB entries used in this study are available in the PDB database under accession codes 6JT1, 6JT2, 3L6J and 5MNW. Other relevant data are available from the corresponding author. Source data are provided with this paper.

## Source data

source data
