# Supplementary material for: Activation mechanism of human soluble guanylate cyclase by stimulators and activators
Source: Nat Commun. 2021 Sep 17;12:5492. doi: 10.1038/s41467-021-25617-0 (PMC8448884; doi:10.1038/s41467-021-25617-0)
Supplement: Supplementary file 1 — supplementary information [file 41467_2021_25617_MOESM1_ESM.pdf]

Supplementary information for

Activation mechanism of human soluble guanylate cyclase by stimulators and activators

Rui Liu#, Yunlu Kang#, Lei Chen*

*To whom correspondence should be addressed: Lei Chen (chenlei2016@pku.edu.cn)

This file includes:

Supplementary Figs. 1-6

Supplementary tables 1-2

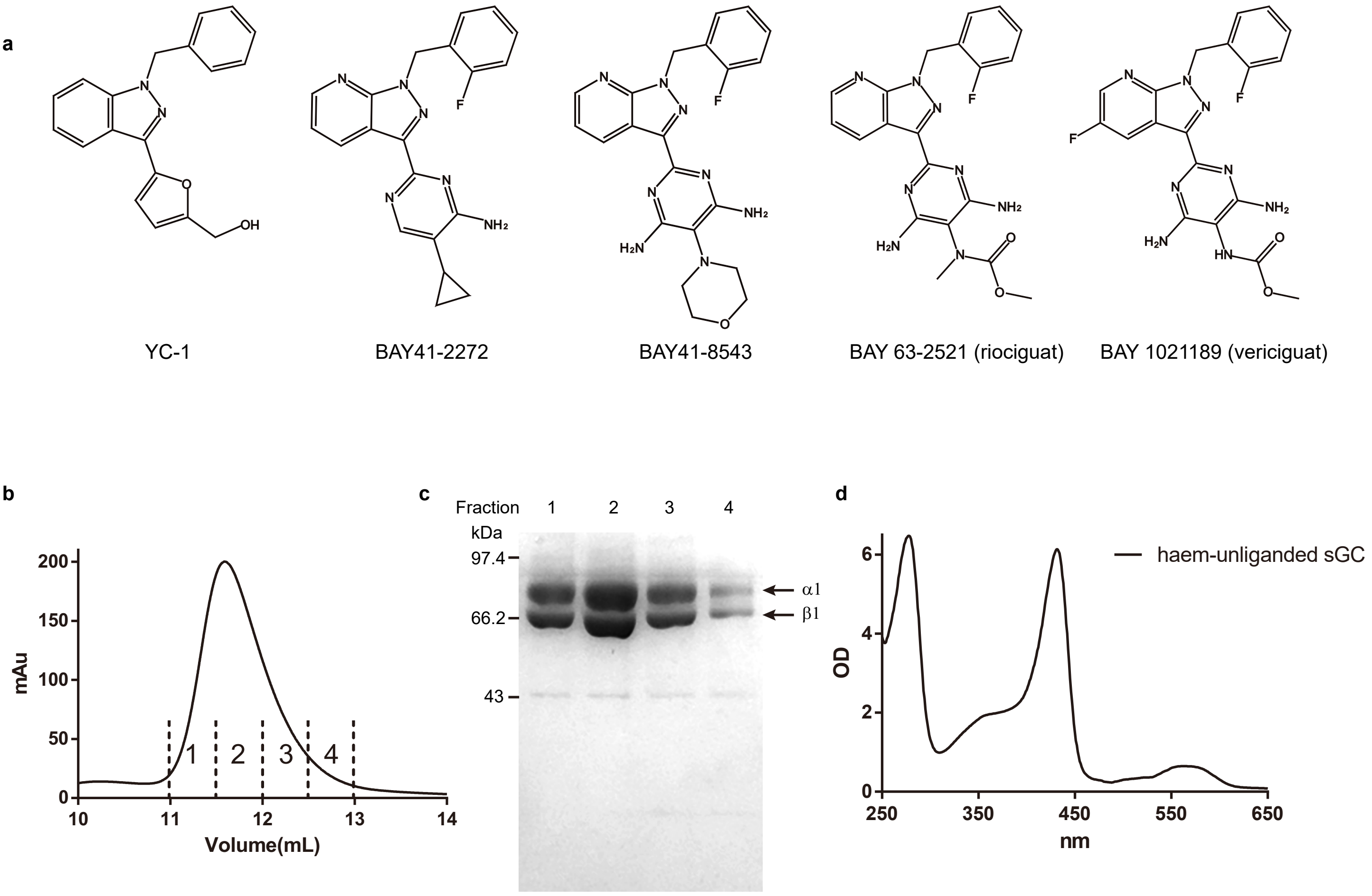

Supplementary Fig. 1| Purification of the wild-type human α1β1 sGC heterodimer protein. a, The chemical structures of several YC-1 class sGC stimulators. **b**, Size-exclusion chromatography of wild-type sGC on a Superdex 200 increase column. Dashed lines indicate the portion of fractions used for cryo-EM sample preparation. **c**, SDS-PAGE of fractions indicated in a. The bands of α1 and β1 subunits are marked by arrows. The experiments were repeated three times with similar results. Source data are provided as a Source Data file. **d**, UV-vis spectrum of wild-type sGC. The position of Soret peaks are indicated by arrows.

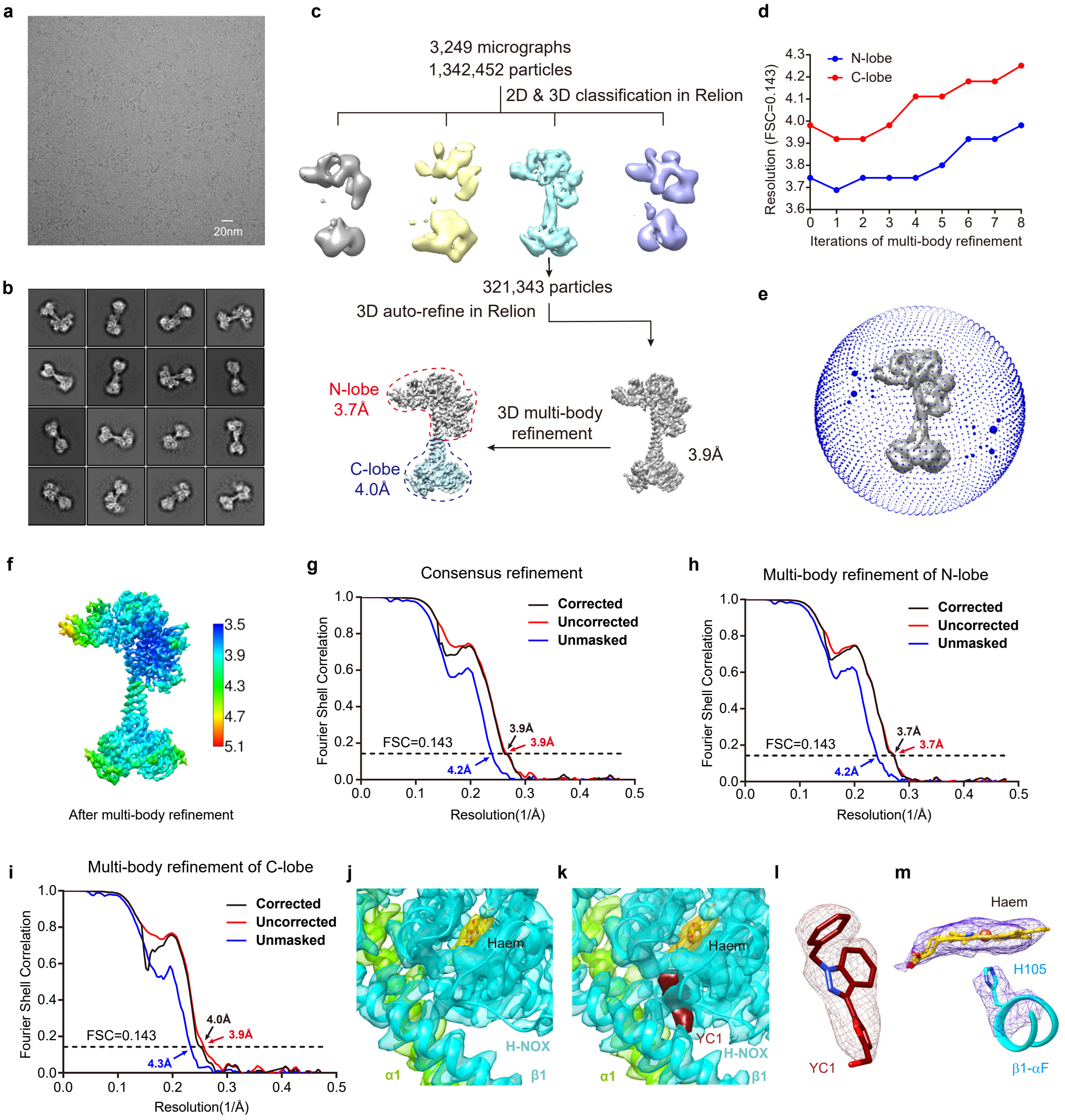

Supplementary Fig. 2 | Cryo-EM data processing of sGC in complex with NO and YC-1. a, Representative raw image of sGC in complex with NO and YC-1. **b**, Representative 2D class average of sGC in complex with NO and YC-1. **c**, The workflow of cryo-EM data processing ofsGC in complex with NO and YC-1. **d**, Resolution estimation of N-lobe and C-lobe maps from each iterations of multi-body refinement (showing dataset of sGC in complex with NO and YC1). Resolution estimations were based on the Fourier shell correlation (FSC) of 0.143 cutoff after correction of the masking effect using post-processing in Relion 3.0. **e**, The angular distribution for the consensus refinement of sGC in complex with NO and YC-1 is indicated by the sizes of spheres. **f**, Local resolution estimation of the composite map of sGC in complex with NO and YC-1 after multibody refinement. **g-i**, Gold-standard FSC curves of sGC in complex with NO and YC-1 after consensus refinement and multibody refinement. Resolution estimations were based on an FSC cutoff value of 0.143. **j-k**, Local density inside the YC-1–binding site of sGC in complex with NO (**j**) and YC-1 (**k**). The YC-1 density is shown in red surface. **l**, Zoom-in view of the YC-1 density contoured at 9σ. **m**, Density map of the β1 H105 side chain and haem group of sGC in complex with NO and YC-1.

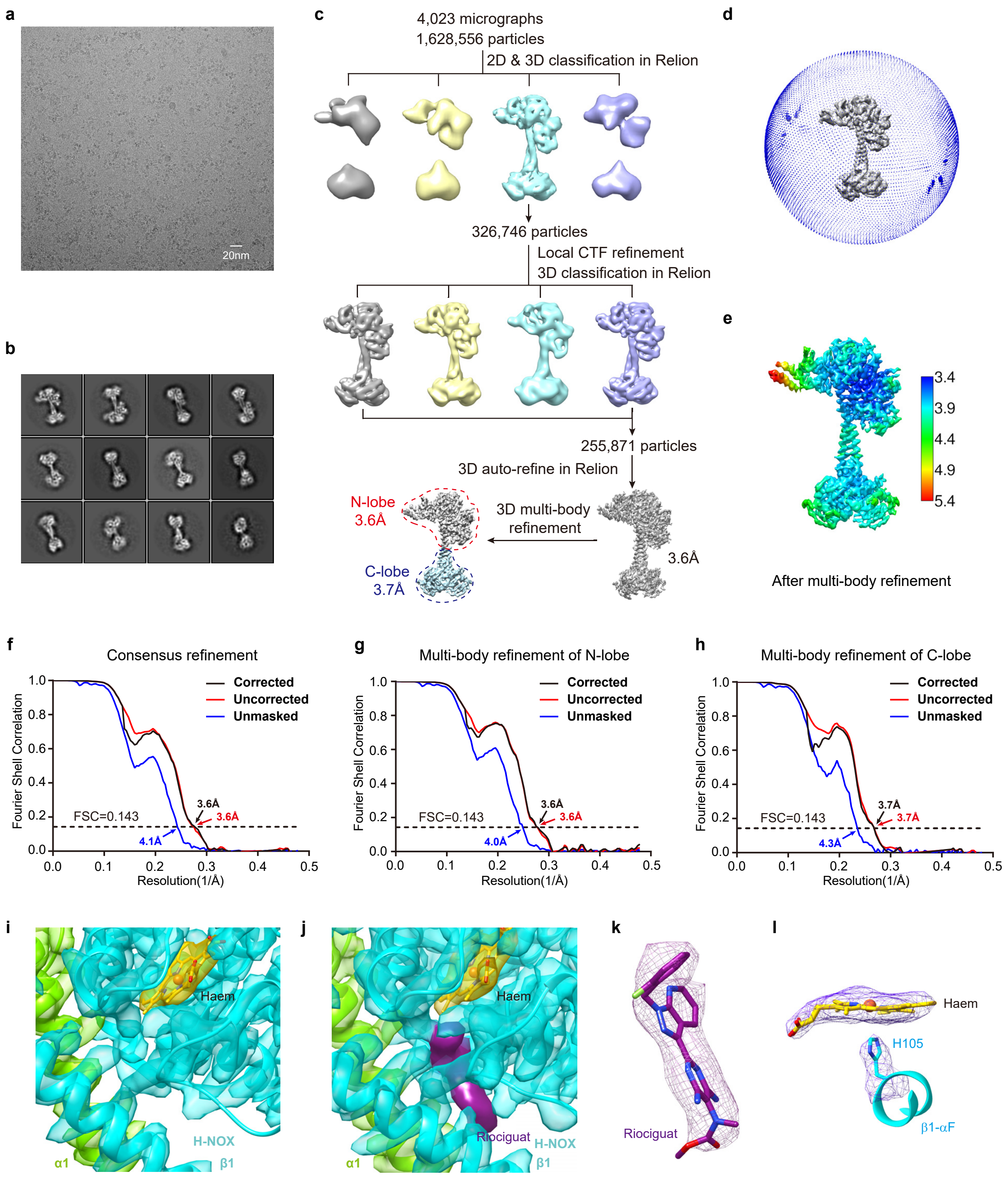

Supplementary Fig. 3 | Cryo-EM data processing of sGC in complex with NO and riociguat. a, Representative raw image of sGC in complex with NO and riociguat. **b**, Representative 2D class average of sGC in complex with NO and riociguat. **c**, The workflow of cryo-EM data processing of sGC in complex with NO and riociguat. **d**, The angular distribution for the consensus refinement of sGC in complex with NO and riociguat is indicated by the sizes of spheres. **e**, Local resolution estimation of the composite map of sGC in complex with NO and riociguat after multibody refinement. **f-h**, Gold-standard FSC curves of sGC in complex with NO and riociguat after consensus refinement and multibody refinement . Resolution estimations were based on an FSC cutoff value of 0.143. **i-j**, Local density inside the riociguat-binding site of sGC in complex with NO (**i**) and riociguat (**j**). The riociguat density is shown in purple surface. **k**, Zoom-in view of the riociguat density contoured at 9σ. **l**, Density map of the β1 H105 side chain and haem group of sGC in complex with NO and riociguat.

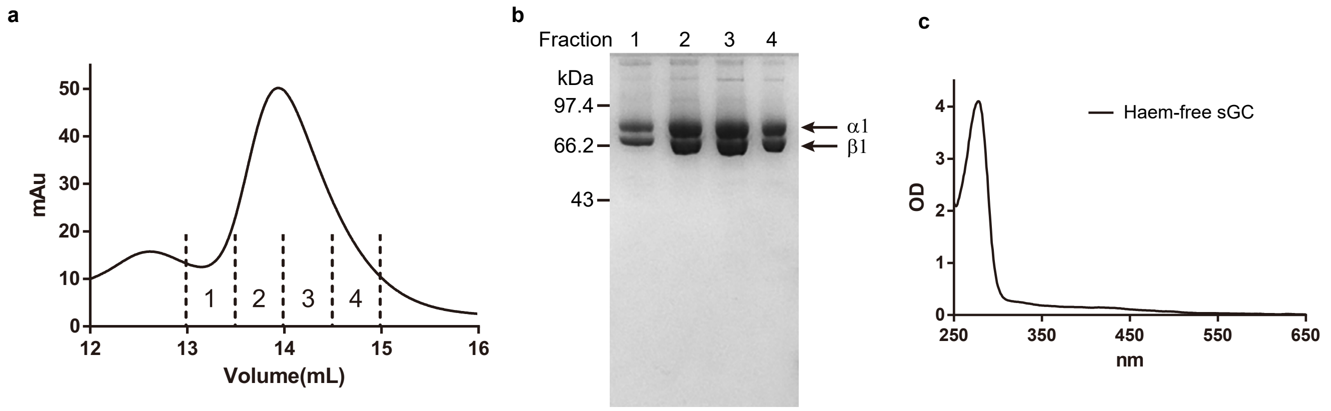

Supplementary Fig. 4 | Purification of the haem-free α1β1 sGC heterodimer protein. a, Size-exclusion chromatography of haem-free sGC on a Superdex 200 column. Dashed lines indicate the portion of fractions used for cryo-EM sample preparation. **b**, SDS-PAGE of fractions indicated in a. The bands of α1 and β1 subunits are marked by arrows. The experiments were repeated twice with similar results. Source data are provided as a Source Data file. **c**, UV-vis spectrum of haem-free sGC suggests the completeness of haem removal.

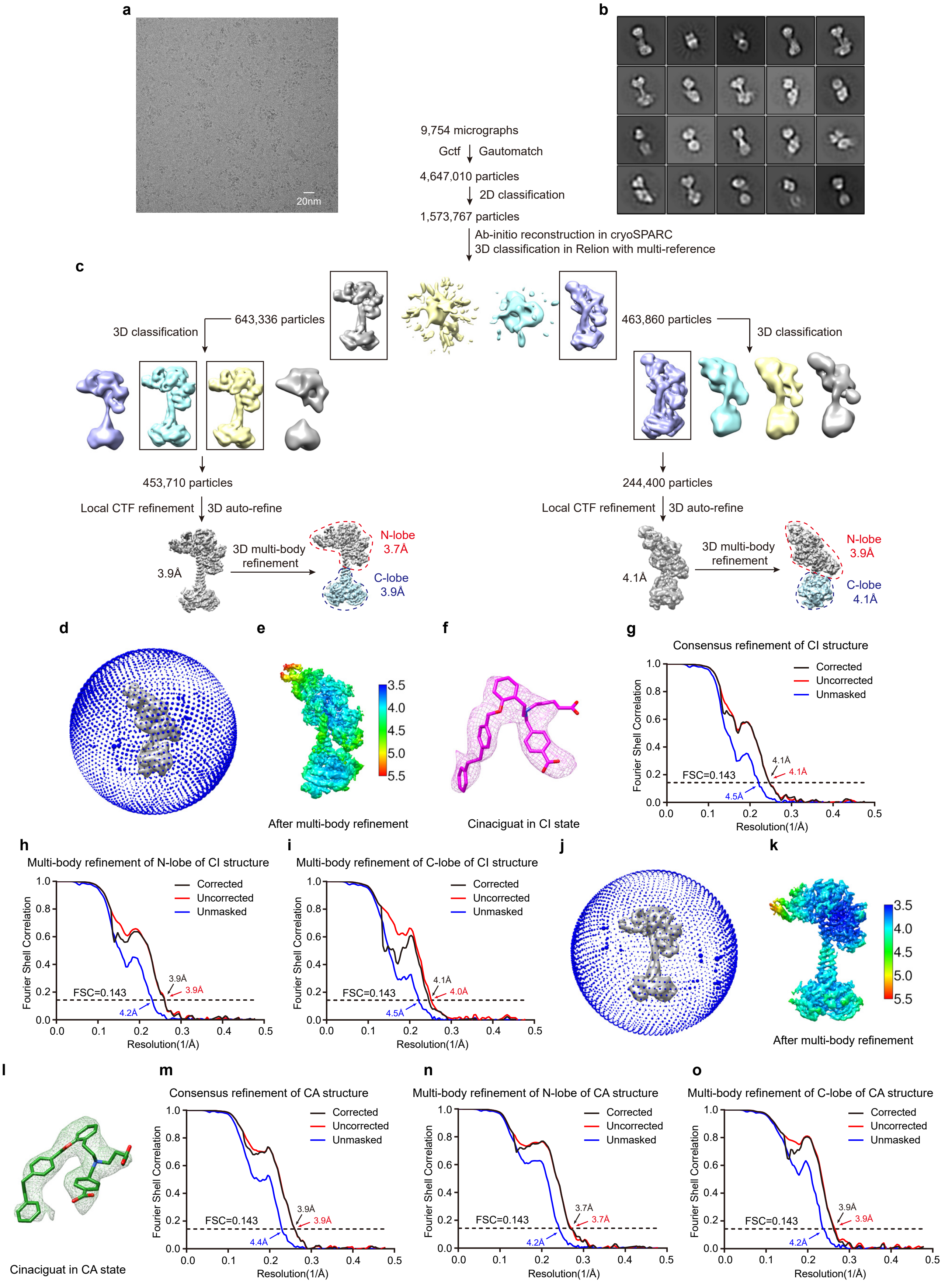

Supplementary Fig. 5 | Cryo-EM data processing of cinaciguat-bound sGC. a, Representative raw image of cinaciguat-bound sGC. **b**, Representative 2D class average of cinaciguat-bound sGC. **c**, The workflow of cryo-EM data processing of cinaciguat-bound sGC. **d**, The angular distribution for the consensus refinement of cinaciguat-bound inactive sGC is indicated by the sizes of spheres. **e**, Local resolution distribution of the composite map of cinaciguat-bound inactive sGC after multibody refinement. **f**, Cinaciguat density of sGC in the cinaciguat-bound inactive state. The density is contoured at 7σ. **g-i**, Gold-standard FSC curves of cinaciguat-bound inactive sGC after consensus refinement and multibody refinement. Resolution estimations were based on an FSC cutoff value of 0.143. **j**, The angular estimation for the consensus refinement of cinaciguat-bound activated sGC is indicated by the sizes of spheres. **k**, Local resolution estimation of the composite map of cinaciguat-bound activated sGC after multibody refinement. **l**, Cinaciguat density of sGC in the cinaciguat-bound activated state. The density is contoured at 9σ. **m-o**, Gold-standard FSC curves of cinaciguat-bound activated sGC after consensus refinement and multibody refinement. Resolution estimations were based on an FSC cutoff value of 0.143.

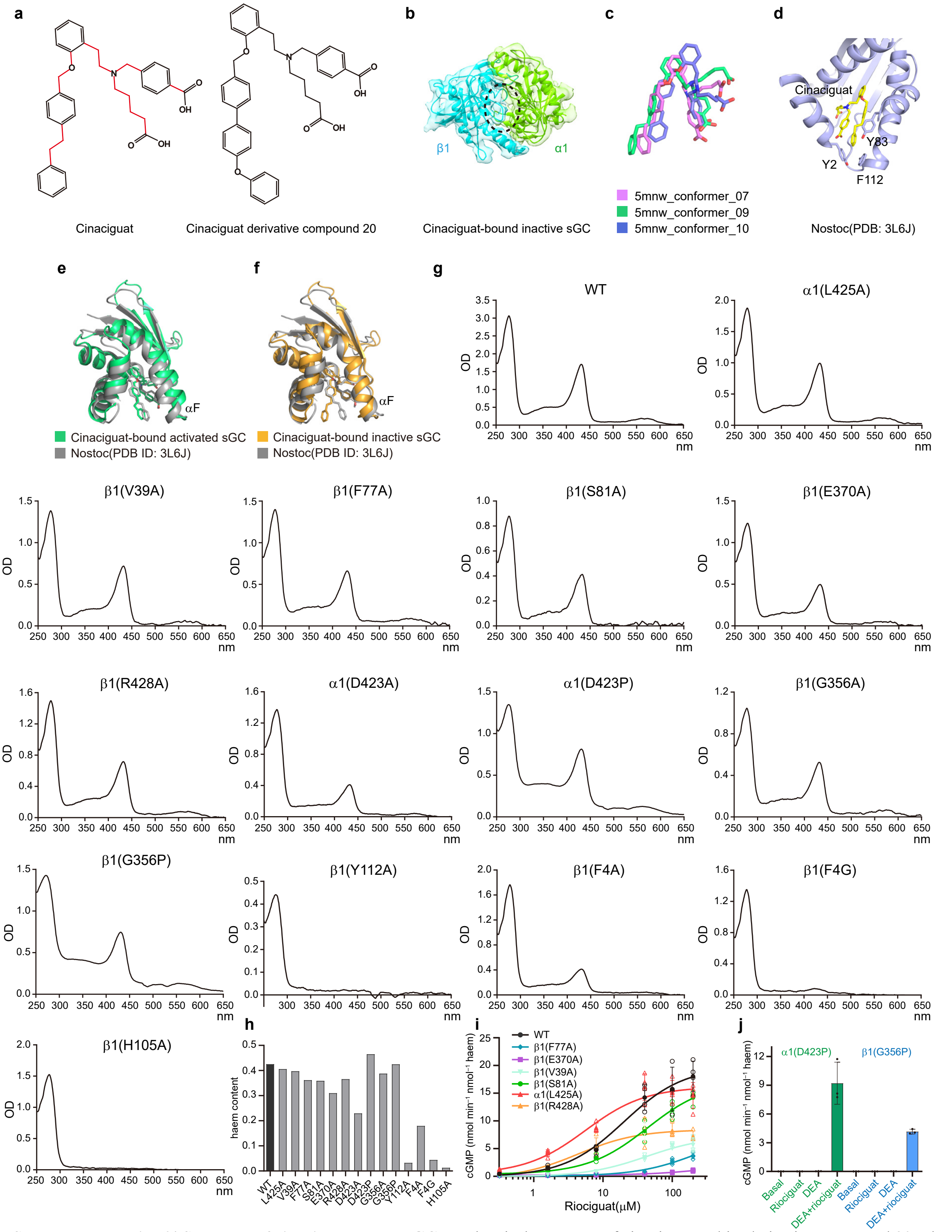

Supplementary Fig. 6 | Structure of cinaciguat-bound sGC. **a**, Chemical structures of cinaciguat and its derivative compound 20. The rotable bonds of cinaciguat are shown in red. **b**, Bottom view of the cryo-EM map of the sGC catalytic module in the inactive state. The density of sGC is shown in transparency. The apo-binding site of GMPCPP is indicated by a dashed line. **c**, Structure comparison of the cinaciguat-bound human β1 H-NOX structure solved by NMR (PDB ID: 5MNW). Cinaciguat of conformers 7, 9, and 10 are shown as sticks. **d**, Cartoon representation of the cinaciguat-binding site of the Nostoc H-NOX domain (PDB ID: 3L6J). **e**, Structural comparison between the C-terminal subdomain of β1 H-NOX of cinaciguat-bound activated sGC (green) and Nostoc H-NOX domain (grey, PDB ID: 3L6J). **f**, Structural comparison between the C-terminal subdomain of β1 H-NOX of cinaciguat-bound inactive sGC (orange) and Nostoc H-NOX domain (grey, PDB ID: 3L6J). **g**, UV-vis spectrum of purified sGC samples used for enzymatic assays. **h**, Haem content of sGC samples corresponding to **g**, which are calculated with molar extinction coefficient at A_{280} and A_{431}. Source data are provided as a Source Data file. **i**, Dose-dependent activation curve of wild type and alanine mutants of sGC in the presence of 1 mM BIC and riociguat at different concentrations ranged from 0.32 mM to 200 mM. Mean ± s.d., n = 3 or 4 independent reactions. The reaction rates were normalized by haem contents. Source data are provided as a Source Data file. **j**, End-point activity assay of sGC proline mutations in the absence or presence of 100 mM DEA or 200 mM riociguat. Mean ± s.d., n = 3 independent reactions. The reaction rates were normalized by haem contents. Source data are provided as a Source Data file.

Supplementary Table. 1
Cryo-EM data collection, refinement and validation statistics

	YC-1-bound state	riociguat-bound state	cinaciguat-bound inactive state	cinaciguat-bound activated state
PDB ID	7D9S	7D9R	7D9T	7D9U
EMDB ID	EMD-30619	EMD-30618	EMD-30620	EMD-30621
**Data collection and processing**				
Magnification	130,000×			
Voltage (kV)	300			
Electron exposure (e⁻/Å^2)	48			
Defocus range (μm)	-1.5 to -1.8			
Pixel size (Å)	1.045			
Symmetry imposed	*C1*			
Initial particle images (no.)	1,342,452	1,628,556	4,647,010	
Final particle images (no.)	321,343	255,871	244,400	453,710
Map resolution (Å)	3.9 (3.7/4.0)*	3.6 (3.6/3.7)*	4.1 (3.9/4.1)*	3.9 (3.7/3.9)*
FSC threshold	0.143	0.143	0.143	0.143
Map resolution range (Å)	250.0-3.7	250.0-3.6	250.0-3.9	250.0-3.7
**Refinement**				
Initial model used (PDB code)	6JT2	6JT2	6JT1	6JT2
Model resolution (Å)	3.6	3.5	3.7	3.6
FSC threshold	0.143	0.143	0.143	0.143
Model resolution range (Å)	250.0-3.6	250.0-3.5	250.0-3.7	250.0-3.6
Map sharpening *B* factor (Å^2)	-189 (-203/-250)*	-184 (-176/-218)*	-166 (-169/-204)*	-183 (-186/-220)*
Model composition				
Non-hydrogen atoms	8,206	8,214	7,451	8,191
Protein residues	1,071	1,071	1,079	1,075
Ligands	5	5	1	4
B factors (Å^2)	120.68	104.75	135.36	119.21
Protein	120.76	104.68	135.39	119.31
Ligand	114.39	110.53	128.44	108.01
R.m.s. deviations				
Bond lengths (Å)	0.003	0.003	0.003	0.003
Bond angles (°)	0.557	0.536	0.551	0.501
Validation				
MolProbity score	1.76	1.66	1.63	1.68
Clashscore	7.91	7.23	6.66	8.35
Poor rotamers (%)	0.00	0.00	0.00	0.12
Ramachandran plot				
Favored (%)	95.30	96.16	96.10	96.47
Allowed (%)	4.51	3.64	3.90	3.43
Disallowed (%)	0.19	0.19	0.00	0.10

* The numbers outside the brackets are from the consensus refinement. Numbers inside brackets are from the multibody refinement (N-lobe/C-lobe).

The parameters for the Cryo-EM data collection, processing and validation of the four sGC structures are listed in the table. The columns which contain the same parameters are combined into one.

Supplementary Table. 2
The potency of riociguat on various sGC constructs in the presence of 1 mM BIC

sGC constructs	Riociguat	
	$LogEC_{50}$[a]	EC_{50} (μM)
WT	1.30 ± 0.14	20.08
α1(L425A)	0.75 ± 0.14	5.59
β1(V39A)	1.64 ± 0.13	43.14
β1(F77A)	-	>100[b]
β1(S81A)	1.64± 0.09	43.97
β1(E370A)	-	>100[b]
β1(R428A)	0.64 ± 0.20	4.35

[a]Data were expressed as $logEC_{50}$ ±SEM, n=4.
[b]The range of fitted EC_{50} was too wide and the value was larger than 100μM at least.

The EC50 of Riociguat on WT and mutated sGC was measured in the presence of 1mM BIC, which is a distal haem ligand. The corresponding curves are shown in Fig. 2f.
